# Antibiotic and Metal Resistance in *Escherichia coli* Isolated from Pig Slaughterhouses in the United Kingdom

**DOI:** 10.3390/antibiotics9110746

**Published:** 2020-10-28

**Authors:** Hongyan Yang, Shao-Hung Wei, Jon L. Hobman, Christine E. R. Dodd

**Affiliations:** 1 College of Life Sciences, Northeast Forestry University, Harbin 150040, China; 2 School of Biosciences, University of Nottingham, Sutton Bonington Campus, Sutton Bonington, Leicestershire LE12 5RD, UK; mwei@jhlbiotech.com (S.-H.W.); Jon.Hobman@nottingham.ac.uk (J.L.H.); Christine.dodd@nottingham.ac.uk (C.E.R.D.); 3 JHL Biotech, Zhubei City, Hsinchu County 302, Taiwan

**Keywords:** antibiotic, metal, resistance, *Escherichia coli*, slaughterhouse

## Abstract

Antimicrobial resistance is currently an important concern, but there are few data on the co-presence of metal and antibiotic resistance in potentially pathogenic *Escherichia coli* entering the food chain from pork, which may threaten human health. We have examined the phenotypic and genotypic resistances to 18 antibiotics and 3 metals (mercury, silver, and copper) of *E. coli* from pig slaughterhouses in the United Kingdom. The results showed resistances to oxytetracycline, streptomycin, sulphonamide, ampicillin, chloramphenicol, trimethoprim–sulfamethoxazole, ceftiofur, amoxicillin–clavulanic acid, aztreonam, and nitrofurantoin. The top three resistances were oxytetracycline (64%), streptomycin (28%), and sulphonamide (16%). Two strains were resistant to six kinds of antibiotics. Three carried the *bla*_TEM_ gene. Fifteen strains (18.75%) were resistant to 25 µg/mL mercury and five (6.25%) of these to 50 µg/mL; *merA* and *merC* genes were detected in 14 strains. Thirty-five strains (43.75%) showed resistance to silver, with 19 possessing *silA*, *silB*, and *silE* genes. Fifty-five strains (68.75%) were resistant to 8 mM copper or above. Seven contained the *pcoE* gene. Some strains were multi-resistant to antibiotics, silver, and copper. The results in this study, based on strains isolated between 2007 and 2010, will aid understanding about the effects of strategies to reduce resistance and mechanisms of antimicrobial resistance (AMR).

## 1. Introduction

The discovery and introduction of antibiotics prior to the Second World War marked a pivotal point in the ability of humanity to combat bacterial infections [1]. For over half a century, antibiotics have been widely used in the animal production industry. They have been used not only for disease prevention and treatment, but also for promotion of growth [2]. It is estimated that half of the total antibiotics produced are used for veterinary purposes [3]. The overuse and misuse of antibiotics stimulated the more rapid emergence and spread of antibiotic-resistant bacteria (ARB) and antibiotic resistant genes (ARGs), reducing their therapeutic potential against human and animal pathogens [4,5]. The occurrence of multidrug-resistant microorganisms has brought emergent threats to human health.

Metals and metalloids have a long history of human usage in medicine and agriculture. Some toxic metal(loid) compounds, such as mercury and arsenic/antimony compounds, were still used as first-line drugs or preferred-choice chemotherapeutics or antimicrobials in the late twentieth century [6]. Some metals, such as copper and zinc, are still used in animal husbandry as antimicrobials and as essential micro-nutrients and growth stimulants [7,8,9]. However, the actual concentration of metals used in feed samples is far higher than that of the growth requirements [10,11]. Overuse and misuse of metals has resulted in their accumulation in the environment, which could promote ARB through co-selection [2,12,13,14]. Metal pollutants have been hypothesized to be notable contributors to the development of ARB and its determinants [15,16,17].

Pork is the most consumed meat globally [18]. There are many steps in the production of pork meat from farm to product such as breeding and finishing, animal transportation, slaughter, cutting, processing, and packing, and each step can be a source of bacterial contamination [19,20,21,22]. In pig slaughterhouses, many microorganisms such as *Salmonella* spp.*, Campylobacter* spp.*, Listeria monocytogenes*, and *E. coli*, which can cause human foodborne disease, have been detected [23,24]. *E. coli* is widely distributed in the environment and can be used to indicate faecal contamination and potential transfer of enteric pathogens [22,25]. Depending on the categories, pathogenic *E. coli* can cause infection with colonisation of a mucosal site, and is associated with evasion of host defenses, toxin production, and others [26,27]. Many acquired pathogenicity genes, such as ST1 and LT in Enterotoxigenic *E. coli*, *eae* in Enteropathogenic *E. coli*, and *astA* in Enteroaggregative *E. coli*, have been described, with some of these genes carried on plasmids. *E. coli* strains that carry some of these pathogenicity genes have been found in pork and pigs at slaughter [28,29,30], and ARB in bacterial isolates from meat is a potential route for transmission into humans. A combination of these virulence factor genes with antibiotic resistance and metal resistance could lead to the development of a serious pathogen. For example, the enterohaemorrhagic *E. coli* O104:H4 strain, which caused an international disease outbreak in 2011, carried the *vtx2a* gene (Shiga toxin 2), but also had the typical enteroaggregative *E. coli* pAA virulence plasmid, which codes the AAF adhesion fimbriae (*astA* gene). This strain was also multidrug resistant and in particular carried a resistance plasmid with genes (TEM-1, CTX-M) for extended-spectrum β-lactamase [31]. In addition, the emergence of the first plasmid-mediated colistin resistance mechanism, *mcr-1*, in *Escherichia coli* from pigs, pork products, and humans has been a major concern and heralded the breach of the last line group of antibiotics, polymyxins, which indicated that coordinated global action in the fight against pan-drug-resistant Gram-negative bacteria is urgently needed [32].

A limited number of studies have examined the influence of copper on ARB of the gut microbiota in pigs [33]; from their systematic review of the literature, the authors concluded that the available data ‘do not allow excluding the possibility of a positive correlation between copper supplementation above requirements and development of antibiotic resistance’. There are, however, very little data on the co-presence of metal and antibiotic resistance in potentially pathogenic *E. coli* entering the food chain from pork.

A commonly applied microbial storage technique is to create a dry, low-temperature, and anoxic environment to inhibit the metabolism of microorganisms. After long-term preservation, the survival rate of microbes should be well maintained and microbial activity should get a rapid recovery [34,35]. The liquid nitrogen (−196 °C) lyophilization method and freezing (−20~80 °C) method are most commonly used [36]. MicroBank^TM^ beads, as a long-term storage method, have been used for bacteria and fungal storage. The method of storage is simple, economical, and practical [36]. Between 2007 and 2010, over one thousand *E. coli* strains were isolated in our laboratory from pigs in slaughterhouses in the United Kingdom and stored using MicroBank^TM^ beads at −80 °C [37]. Eighty-one strains were shown to carry the virulence gene *astA* associated with enteroaggregative *E. coli* (EAggEC) strains [37], and thus potentially capable of causing human gastrointestinal disease. We have now further examined these strains to obtain systematic data on their phenotypic and genotypic resistances to antibiotics and metals and to evaluate their co-occurrence.

## 2. Results

### 2.1. Isolate Recovery and EAggEC Genes Re-Confirmation

All eighty-one putative EAggEC strains were streaked on Brain Heart Infusion (BHI) agar from stock; eighty strains were recovered successfully. All 80 strains gave blue colonies typical of *E. coli* on Tryptone Bile X-glucuronide (TBX) agar. All strains were oxidase negative. Seventy-seven strains were positive for the indole test, confirming these were *E. coli*. The EAggEC gene *astA* was found in 70 strains and these isolates were studied further.

### 2.2. Resistance Prevalence to Antimicrobials

**Figure 1:**
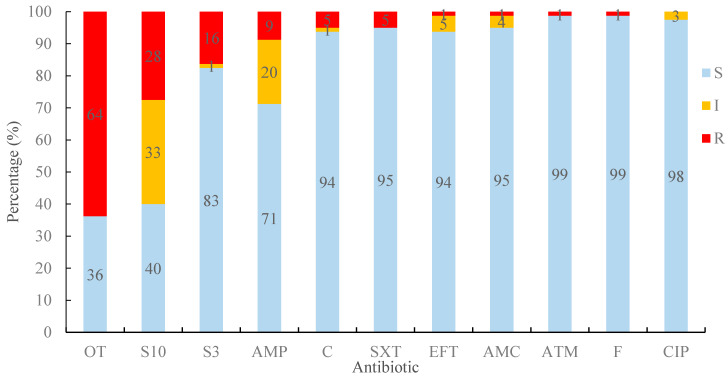
Percentage sensitivity to 18 antibiotics for 80 *E. coli* strains. Blue bars indicate sensitive (S), yellow bars indicate intermediate sensitivity (I), and red bars indicate lack of sensitivity (R), using Clinical and Laboratory Standards Institute (CLSI) definitions [39,40,41]. For antibiotic abbreviations, see Table 3.

Strains were tested for resistance to a number of classes of antibiotics commonly used in human medicine that have also been used in animal husbandry, including *β*-lactams, and for which resistance can occur in *E. coli* from animal sources [38]. The phenotypic resistance results are shown in Figure 1. Of 18 antibiotics, resistances to oxytetracycline (OT), streptomycin (S10), sulphonamide (S300), ampicillin (AMP), chloramphenicol (C), trimethoprim–sulfamethoxazole (SXT), ceftiofur (EFT), amoxicillin–clavulanic acid (AMC), aztreonam (ATM), and nitrofurantoin (F) were detected. The top three antibiotic resistances were OT (64%), S10 (28%), and S300 (16%), respectively. There were some strains that showed intermediate resistance, such as to S10 (33%), AMP (20%), EFT (5%), AMC (4%), and CIP (3%). Thirteen percent of isolated strains were sensitive to all antibiotics and 9% of isolated strains showed intermediate sensitivity to all antibiotics; 78% showed resistance to at least one antibiotic.

**Figure 2:**
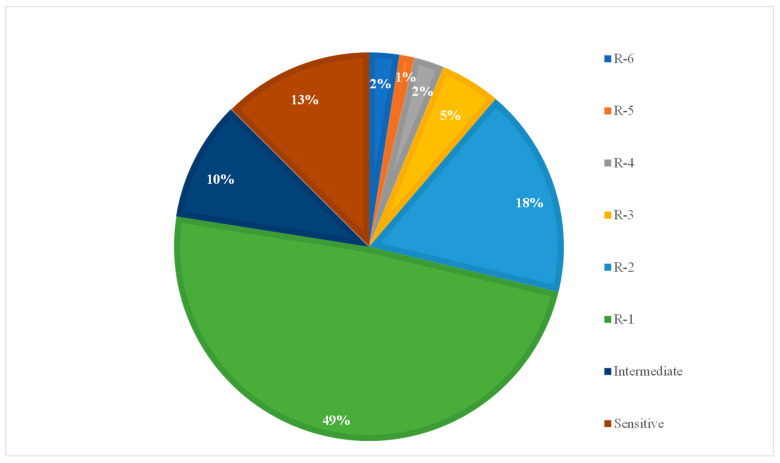
Percentage of *E. coli* resistant to different numbers of antibiotics. R-1~R-6: resistant to 1~6 antibiotics, respectively.

Some strains were resistant to six antibiotics (Figure 2 and Table 1). All strains were sensitive to ceftazidime (CAZ), cefotaxime (CTX), cefquinome (CFQ), imipenem (IPM), and enrofloxacin (ENR). We also examined resistance to colistin sulfate by agar dilution. The results showed that all strains were sensitive to 8 mg/L colistin sulfate (Table S1).

As phenotypic resistance was shown to some *β-*lactamases by seven strains, the presence of the antibiotic resistance genes, *bla*_SHV_, *bla*_TEM_, *bla*_OXA-1_, and *bla*_CTX-M_, was examined by specific polymerase chain reaction (PCR). The results specifically showed that three strains, No. 4, No. 7, and No. 61, carried the *bla*_TEM_ gene (Table 2). According to the phenotypic results for antibiotic resistances, these three strains were the top three resistant strains, which were resistant to six, six, and five antibiotics, respectively. All three strains were resistant to AMP, but only strain 61 showed resistance to AMC, which can be associated with carriage of a plasmid borne *bla*_TEM_ [42], and none showed typical extended spectrum β-lactamase (ESBL) resistance. Strains were also examined for the presence of *mcr-1.* No strains were detected carrying the *mcr-1* gene, confirming the phenotypic result of colistin sulphate sensitivity.

### 2.3. Resistance to Mercury

Various methods for testing for metal resistance exist in the literature. The levels at which toxicity is shown is influenced by many factors, particularly medium composition. Therefore, in this study, known resistant and sensitive strains were included for comparison and the levels at which strains grew or did not grow were compared with these to determine if the test strains were sensitive or resistant.

**Figure 3:**
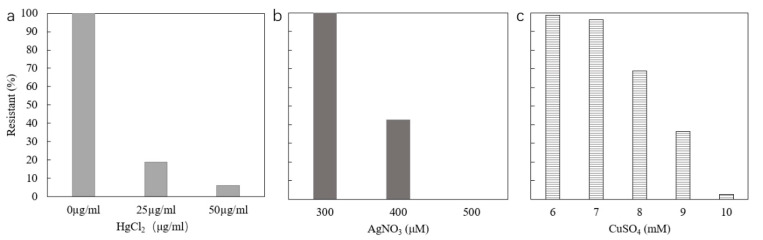
Metal phenotypic resistances of *E. coli* isolates. (**a**) *E. coli* strain J53 was completely inhibited at 25 μg/mL HgCl_2_; (**b**) silver resistance, *E. coli* strain J53 was completely inhibited at 400 μM AgNO_3_; (**c**) copper resistance, *E. coli* strain ED8739 was completely inhibited at 7 mM CuSO_4_.

Phenotypic mercury resistance was tested by growth of strains on media with different concentrations of HgCl_2_. The level of HgCl_2_ in Luria-Bertani (LB) agar that completely inhibits the growth of the *E. coli* sensitive control strain is 25 μg/mL. For the tested strains, 15 (18.75%) were resistant to 25 µg/mL mercury and 5 (6.25%) were resistant to 50 µg/mL mercury (Figure 3a). Especially for strain 38, resistance was shown as same as the positive control (50 µg/mL) (*E. coli* J53 pMG101).

The specific primers of *merA*, *merC*, and *intl1* genes were used to look for genetic elements associated with mercury resistance and carriage of Tn*21*-like transposable elements carrying an integron. The *merA* and *merC* genes were detected in 14 of the 80 (17.5%) strains and *intl1* was detected in 4 strains (5%). Two strains, 10 and 14, contained both *merA*, *merC*, and the *intl1* genes (Table 2), indictive of carrying a transposon. Combined with phenotypic resistance results, these 14 strains containing *merA* and *merC* genes all showed resistance to 25 μg/mL mercury. Strain 38, containing *merA*, *merC,* and *astA*, was even resistant to 50 μg/mL.

### 2.4. Resistance to Silver

LB agar without salt with different concentrations of AgNO_3_ was used for detecting phenotypic silver resistance. The results showed all strains could grow at 300 µM AgNO_3_. At 400 µM AgNO_3_, *E. coli* sensitive control strain was completely inhibited. Of the tested strains, 35 strains (43.75%) showed silver resistance to 400 µM AgNO_3_ (Figure 3b). All strains were sensitive to 500 µM AgNO_3_. Of the 35 phenotypically resistant strains, 19 strains possessed *silA*, *silB*, and *silE* genes (Table 2).

### 2.5. Resistance to Copper

In this study, *E. coli* ED8739 and *E. coli* ED8739 pRJ1004 were used as negative and positive controls, respectively. The results showed that *E. coli* ED8739 could grow at 6 mM CuSO_4_ or below, but could not grow at 7 mM copper. However, *E. coli* ED8739 pRJ1004 expressed extremely strong resistance to copper, being resistant to 20 mM CuSO_4_. Compared with *E. coli* ED8739, most strains showed resistance to only 7 mM copper (Figure 3c). Fifty five strains (68.75%) were resistant to 8 mM copper, twenty nine (36.25%) strains were resistant to 9 mM copper, and two strains were resistant to 10 mM copper. Over 10 mM, no strains showed resistance.

At the same time, we examined all strains for carriage of *pcoE*. The *pcoE* gene was detected in seven strains (Table 2). Two strains (No. 50 and No. 77) with the *pcoE* genes showed phenotypic resistance to 8 mM and 9 mM copper, while one strain (No. 78) showed resistance to 10 mM (Table 2). The other four strains (No. 14, No. 63, No. 74, and No. 75) containing the *pcoE* gene did not show resistance to 8 mM copper, only to 7 mM CuSO_4_, slightly above the basal copper tolerance of laboratory *E. coli* strains (Table 2 and Table S1). Most other strains phenotypically copper resistant at 8 mM did not contain a *pcoE* gene (Table S1).

## 3. Discussion

Antimicrobial resistance (AMR) has been characterized as a global public health crisis that must be managed with the utmost urgency [43]. The evolution of AMR is a complex, multifactorial issue [44]. The lack of understanding for the potential dangers of inappropriate use, as well as the widespread access to antimicrobials, have led to the current AMR crisis [45]. Several strategies, such as antimicrobial stewardship programs, preventing the misuse of antibiotics, and promoting the discovery of new antimicrobial agents, have been developed in recent years to slow this progression [44]. Antimicrobial resistance surveillance at different periods will aid people to know about the effects of these strategies and pathologic mechanisms of AMR. To the best of our knowledge, this is the first study to examine the prevalence of the co-occurrence of both antibiotic resistance and metal resistance in *E. coli* from slaughterhouses in the United Kingdom.

In this study, we focused on AMR of *E. coli* strains isolated from pig slaughterhouses in 2007, 2009, and 2010. We used the strain stocks stored on Microbank^TM^ beads at −80 °C. This method is often used for long-term storage of microorganisms [36,46,47]. In this study, of the 81 strains, which were stored in Microbank^TM^ in 2009 and 2010, 80 live strains were recovered, giving a recovery rate of 98.8%, which indicated that Microbank^TM^ could be used to store *E. coli* for at least seven years. However, during storage, some characteristics appeared to have changed. For example, when originally isolated, all strains were detected with the plasmid borne *astA* gene, which is found among certain categories of diarrhoeagenic *E. coli* [48]. After storage, only 70 strains were detected carrying the *astA* gene, suggesting plasmid loss on recovery. Three strains were indole negative, although originally characterised as indole positive. These results showed that long-term storage may lead to the instability of some *E. coli* strain characteristics.

From phenotypic results, 78% of analysed strains were resistant to between one and six antibiotics. Multi-drug resistance prevalence was 28.8%, which was similar to reports from other places at the same time. For instance, Sheikh et al. from Canada (with pork, beef, poultry, and turkey samples) and Llorente et al. from Buenos Aires (with beef samples) reported a multi-drug resistance prevalence of 28.1% and 27.8%, respectively [49,50]. From the Tadesse et al. review from 1950 to 2002 (of human and food samples from beef, pork, poultry), a prevalence of multi-drug resistance of 59.1% in beef and 53.7% in pork was found [51]. The results in the current study showed that the multi-drug resistance prevalence in pigs in the United Kingdom between 2007 and 2010 was very low. In contrast, recent investigations in Tamaulipas, Mexico showed that the prevalence of antimicrobial resistance amounted to 92% in retail samples of beef and pork [30].

Tetracyclines, as a group of antibiotics, are a cause for special attention because large quantities are applied in the therapy in human and animal infections as well as in some countries in animal feed as growth promoters. However, only a small amount of tetracyclines can be metabolized or absorbed by humans and animals. Residues of these antibiotics are accumulated in the environment and may lead to the occurrence of resistant species [52]. Oxytetracycline (OT), belonging to the tetracycline group of antibiotics, is a broad-spectrum antibiotic, active against a wide variety of bacteria [53]. In this study, of all resistances, OT resistance was predominant (n = 51; 64%), with some strains showing only tetracycline resistance, but others showing multi-drug resistance (Figure 1 and Table 1). Similar results were reported in ready-to-eat meat (66.7%) [54], pork (43.8%) [55], beef (74%) [56], and chicken (97%) [57]. The AMR prevalence in Enterobacteriaceae isolates from slaughtered pigs has been shown to be 31.8% in *Salmonella* in 2004–2005 in Ethiopia [58] and 56% in 2013–2014 in Italy [59]; 22% of *E. coli* in 2007 in Denmark [60]; and 78.2% in *Yersinia enterocolitica* in 2013–2014 in Italy [61]. In this current study, 64% of all *E. coli* strains showing resistance was higher than that in Denmark in 2007. In another report, for *Enterococcus faecium* and *E. faecalis* isolated from pigs, the resistance level of 82% in the United Kingdom was higher than the average level (67.4%) in the European Union (EU) in 2013–2014 [62]; the reason for this was unclear. A recent report on resistance in wild pigs showed that, of 115 *E. coli* strains, none were resistant to OT. The abundant prevalence of tetracycline resistance represents a useful marker to monitor antimicrobial-resistant bacteria [63,64]. These results of OT resistance may represent a genetic archaeology of the use of veterinary antimicrobials [38]. Streptomycin (S10) (n = 26; 33%) and sulfonamide (S300) (n = 13; 16%) were the other two antibiotics to which a higher percentage of all strains were resistant in this study. This result was similar to previous reports [55,65,66].

The *β*-lactams are the most widely used class of antimicrobials worldwide, representing over 65% of the global market [67]. From the late 1990s, multidrug-resistant Enterobacteriaceae (mostly *E. coli*) that produce extended spectrum β-lactamases (ESBLs) have emerged within the community setting as an important cause of urinary-tract infections [68]. Extended spectrum β-lactamases (ESBLs), one group of β-lactamase, have the ability to hydrolyse and cause resistance to various types of the newer β-lactam antibiotics, including the extended-spectrum (or third-generation) cephalosporins (e.g., cefotaxime, ceftriaxone, ceftazidime) and monobactams (e.g., aztreonam), but not the cephamycins (e.g., cefoxitin and cefotetan) and carbapenems (e.g., imipenem, meropenem, and ertapenem) [69]. In this study, we have also detected phenotypic resistances to *β*-lactams such as ampicillin (AMP, 9%), amoxicillin–clavulanic acid (AMC, 1%), ceftiofur (EFT, 1%), and aztreonam (ATM, 1%).

For the genotypic detection of β-lactam resistance, PCR amplification of the *bla*_TEM_, *bla*_SHV_, and *bla*_CTX-M_ genes is often used because it is straightforward and cost-effective [68] and, in one report, the *bla*_TEM_ gene was detected as the most prevalent resistance gene in isolates from poultry, beef, and pork in Czech Republic [55]. In this study, the presence of *bla*_TEM_, *bla*_SHV_, and *bla*_CTX-M_ genes was determined. The result showed that the TEM gene existed in three strains (Table 2). Between 2007 and 2010, a large number of ESBL-containing resistant strains were identified in the EU [70,71]. Different results in our study might be because of differences in the antibiotics used in animal husbandry, which may influence resistance carriage. While some characteristics of strains changed during storage, particularly the loss of the *astA* gene, which is usually plasmid carried, it could be suggested that resistance plasmids had also been lost. However, the loss of *astA* was seen in only 12.5% of strains, thus plasmid loss is unlikely to account for the low level of ESBL resistance seen here. The *bla*_TEM_ gene encodes the production of β-lactamase enzymes that hydrolyze the β-lactam ring and inactivate those *β*-lactams [72]. Beta-lactamases are the principal mechanism of bacterial resistance to beta-lactam antibiotics. AmpC beta-lactamases and/or extended-spectrum beta-lactamases (ESBLs) are of particular concern for the prevalence of multidrug-resistant Gram-negative bacteria [73]. In *E. coli*, ESBLs were plasmid-encoded [74], while AmpC beta-lactamase production was chromosomally mediated [73]. Combined with the result in this study, we deduced that the β-lactam resistance seen in the other strains may be conferred by upregulation of the chromosomal *ampC* gene found in all *E. coli* [73], which concurs with the results seen in other studies [38].

According to the phenotypic results, two of the three strains (No. 4 and No. 7) containing the TEM gene showed multi-drug resistance to six antibiotics (AMP, S10, OT, S300, SXT, and C). The other one (No. 61) was resistant to five antibiotics (AMP, AMC, OT, S300, and SXT) (Table 2). However, none of these strains showed typical phenotypic ESBL resistance patterns associated with carriage of the TEM gene. The presence of the *bla*_TEM_ gene might be associated with the co-occurrence of genes for resistance to AMP, OT, S300, and SXT. At the same time, strains No. 4 and No. 7 contained the *intl1* gene (Table 2). According to the report of Fernandez-Alarcon, et al. [75], IncA/C plasmids carrying *bla*_TEM_ and *intl1* and genes conferring resistance to gentamicin, kanamycin, streptomycin, trimethoprim/sulfamethoxazole, sulfoxazole, chloramphenicol, and tetracycline have been described. In this study, whether strains No. 4 and No.7 contain IncA/C plasmids needs to be further researched.

Apart from antibiotics, a similar issue of resistance also exists for metals. Metals also exhibit antimicrobial features. Even when no antimicrobial compounds are used, certain metals can maintain or even increase bacterial resistance against certain agents [76]. In medicine and agriculture, the most commonly used metals have probably been mercury (Hg), copper (Cu), silver (Ag), arsenic (As), and antimony (Sb) [6]; zinc and copper are frequently used as feed supplements for pigs. In this study, we examined *E. coli* resistance to Hg, Cu, and Ag. For Hg, 15 strains (18.75%) were phenotypically resistant to 25 µg/mL HgCl_2_. Of these, 14 strains (93.3%) contained both *merA* and *merC* genes (Table 2). The mercuric ion reductase (MerA), an enzyme that catalyzes the conversion of the thiol-avid Hg (II) to volatile, uncharged Hg (0) that lacks significant affinity for any liganding functional groups, is the heart of the mercury resistance (HgR) mechanism [77]. MerC is an operon-encoded transmembrane protein, particularly found in Tn*21*-family transposons, which pumps ionic mercury into the cytosol [78]. MerC may be needed under conditions of very high Hg (II) exposure [79] and is characteristic of Tn*21*-family transposons. *Intl* is a gene encoding an integrase, which is a necessary component of an integron, and many class 1 integrons (*intl1*) carrying antibiotic resistance cassettes are found on Tn*21*-like transposons [80]. In this study, there were two strains possessing *merA*, *merC,* and *intl1* genes (Table 2). The results indicated that a Tn*21*-like transposon is likely to be present in these two strains.

In this study, to test bacterial silver resistance, silver concentrations of 0~600 μM were set up according to previous research [81]. The results showed that the sensitive strain *E. coli* J53 can grow up to 300 μM of AgNO_3_, while the resistant strain *E. coli* J53 with plasmid pMG101 (J53 pMG101) continues to grow up to 600 μM. Gupta et al. reported that *E. coli* J53 could grow up to 100 μM of AgNO_3_ [81]. Although these differences in resistance levels shown between two studies are possibly due to experimental conditions and operators, resistance at 400 μM of AgNO_3_ was significantly correlated to the presence of genotypic resistances genes (*r* = 0.6), and thus could be taken as the silver concentration that denotes phenotypic resistance. The genes for Ag+ resistance are a cluster of seven genes (and two open reading frames of unknown function), which is organized into three divergently transcribed units [82]. SilE is a periplasmic metal-binding protein that has been purified and measured for Ag^+^-binding properties and is highly expressed when silver ions are present. SilS and SilR are a presumed two-component membrane sensor and transcriptional responder, and SilCBA and SilP are a presumed Ag^+^ efflux chemiosmotic cation-proton antiporter and a P-type ATPase, respectively. In this study, three Ag^+^ resistance genes *silA*, *silB*, and *silE* were examined. The results showed that 23.75% of strains possessed these three genes and expressed phenotypic silver resistance, which may suggest that silver compounds have been widely used as antimicrobials [83]. Of these 23.75% of strains, there were eight strains that also possessed mercury-resistance genes (Table 2). That means these strains possess multi-metal resistances. In addition, there were another 20% of strains that showed phenotypic resistance without *silA*, *silB,* and *silE* genes.

Copper is an essential metal to aerobic forms of life, being involved in donating or accepting electrons in redox-active enzymes, or in the electron transport chain [6,84]. It is commonly used in the farming of pigs, but the actual concentration of copper in the feed is 3–9 times higher than that of the growth requirements [10]. In Europe, copper sulphate in pig feed is permitted at 125–250 mg/kg feed (equivalent to 0.78–1.56 mM) and has been shown to stimulate the growth rate of piglets and, at these levels, is known to modify the gastrointestinal microbiota composition [85]. In this study, three strains were resistant to more than 8 mM copper and one of them (No. 78) was resistant to 10 mM copper (Table 2). However, the phenotypic results of most strains for copper resistance did not match the genotypic ones as seven strains (8.75%) possessed the *pcoE* gene, which is an indicator for carriage of the *pco* copper resistance system, but did not show copper resistance at the level that the positive control (*E. coli* ED8739 pRJ1004) demonstrated (Figure 3c). This result may be due to copper resistance being conferred after a succession of events from a combination of both chromosomal and plasmid-encoded genes [86], or that an incomplete *pco* system is present. The *pco* and *sil* operons have regularly been found together in an identical arrangement in plasmids, but also on the chromosome [6]. In this study, we further found that all strains possessing the *pcoE* gene also contained the *sil* genes.

Noticeably, one strain (No. 14) containing *mer*, *intl1*, *sil*, *pcoE*, and *astA* genes was detected (Table 2). This strain was phenotypically resistant to mercury (25 µg/mL), silver (400 μM), and antibiotics (oxytetracycline and sulfonamide). That means there were co-resistant strains in the food chain. The result also indicated there are high-risk, multi-drug (metal and antibiotics) resistant strains in the food chain that carry genes for potential pathogenicity. Fortunately, no phenotypic copper resistance was detected in this strain.

It is of particular concern that exposure to heavy metals could favor co-selection of antimicrobial resistances [87]. The determination of correlations occurring between antibiotic and heavy metal resistances is considered an important tool to gain new insight into the not yet fully characterized mechanism(s) leading to co-selection [88]. In this study, correlation of metal resistance and antibiotic resistance presence was examined (Table 2). Oxytetracyline was common in all metal resistant strains; 13 of 19 strains with silver resistance showed OT resistance and 14 of 14 strains with mercury resistance showed OT resistance. However, OT resistance was also common in non-metal resistant strains and, for the 80 strains, the Pearson correlation coefficient of OT with silver was *r* = 0.054, showing no specific correlation; for OT with mercury, it was *r* = 0.34, showing weak correlation. However, for all silver resistant strains, OT resistance with silver was *r* = 0.79 and that of OT with mercury was *r* = 1. The results showed that a correlation existed for OT and silver and a strong correlation existed for OT and mercury. These results also indicate co-selection between antibiotic and metal might exist in *E. coli* isolates in this study.

The strains used in this study were collected between 2007 and 2010. There have since been very significant reductions in antibiotic usage in pig husbandry in the United Kingdom, including in use of human critical antibiotics such as fluoroquinolones, 3/4 generation cephalosporins, and colistin [89], and there are plans to remove zinc as a supplement in pig feed in the EU. These data, therefore, provide an historic baseline against which the results of these changes in antimicrobial usage can be measured. The retention of resistances to antimicrobials no longer in active use may demonstrate a genetic archaeology of the use of veterinary antimicrobials and provide a better understanding of the drivers for their presence.

## 4. Materials and Methods 

### 4.1. Isolate Recovery and Re-confirmation

Brain Heart Infusion agar (Sigma, UK) was used to recover the isolates stored in Microbank (Pro-Lab, UK) beads at −80 °C. The inoculated plates were incubated at 37 °C for 24 h. LB agar (Sigma, UK) was used to cultivate the isolates. Growth on TBX agar (Sigma, UK) was used to re-confirm the isolates as *E. coli*. An oxidase test was performed using a strip (MB0266A, Oxoid, UK) as directed by the manufacturer. An indole test was performed using the RapID Spot Indole Reagent (R8309002, SLS, UK). Several drops of Indole Spot Reagent were placed on a piece of filter paper. With an inoculating loop a portion of an 18–24 hour growth isolated colony was picked up and rubbed onto the reagent saturated area. A positive reaction was denoted by the appearance of a blue to blue-green colour change on the bacterial smear within 10 seconds. Negative reactions remained colourless or light pink.

### 4.2. Antibiotic Sensitivity Tests

The disc diffusion antibiotic sensitivity tests were carried out according to Clinical and Laboratory Standards Institute (CLSI) guidelines [40,41]. Four or five bacterial colonies were taken from LB plates, which had been incubated overnight at 37 °C. The colonies were suspended in 5 mL of Mueller–Hinton broth (Oxoid, UK) to a 0.5 McFarland standard concentration. Then, 100 μL of the culture was pipetted onto the surface of a 25 mL Mueller–Hinton agar plate (Oxoid, UK), and the inoculum was distributed by spreading using a sterile swab. The plates were left to dry at room temperature (for no more than 15 min), and the antibiotic discs were applied using a disc dispenser (Pro-lab, UK). The plates were then incubated at 37 °C for 18–24 h, and the results were recorded by measuring the inhibition zone diameter across the disc and interpreted according to standard measurement tables [40,41]. The seventeen antibiotics used in the tests are listed in Table 3; discs were supplied by Oxoid (UK), except for Cefquinome (Bioconnection, UK), and the quality control strains used were *E. coli* ATCC 25922 (ESBL negative) and *E. coli* NCTC 13353 (ESBL positive CTX-M-15). For phenotypically colistin-resistant isolates, the agar dilution method was used according to the CLSI procedure [39]. Isolates showing growth above the concentration of 8 mg/L colistin were regarded as resistant [90].

### 4.3. Metal Sensitivity Tests

Ag+ resistance was assayed on LB agar plates without added NaCl [72] with the addition of AgNO_3_ (Sigma Aldrich, 209139) at concentrations of 0 μM, 100 μM, 200 μM, 300 μM, 400 μM, 500 μM, and 600 μM. Exponential-phase cultures were streaked on plates and incubated at 37 °C for 16 h before recording growth [91]. *E. coli* strain J53 and *E. coli* strain J53 pMG101 were used as negative and positive control, respectively [81]. Phenotypic mercury resistance was tested by growth on LB agar supplemented with HgCl_2_. Concentrations of 0, 25 μg/mL, and 50 μg/mL were prepared by the addition of 50 mg/mL HgCl_2_ to sterilised LB agar. Strains were streaked on the plates and incubated at 37 °C for 16 h before recording growth. For copper sensitivity tests, LB agar plates were supplemented with CuSO_4_ (Sigma Aldrich, C1297, UK) (0, 2, 4, 5, 6, 7, 8, 9, 10, 11, 12, 14, 16, 18, and 20 mM). The pH of the agar was adjusted to pH 7.2 [92]. *E. coli* ED8739 [93] and *E. coli* ED8739 pRJ1004 (plasmid with *pco* genes) [94] were used as negative and positive controls, respectively.

### 4.4. PCR Detection of Genetic Elements Carrying Metal and Antibiotic Resistance Genes

Bacterial DNA was extracted by dispersing two colonies of *E. coli* from an overnight culture on LB agar into 100 μL of sterile 1 × TE buffer (10 mM Tris-Cl, 1 mM EDTA buffer, pH 7.6). The suspension was heated to 100 °C for 30 min to rupture bacterial cells (Eppendorf Thermomixer Comfort, Germany) and then centrifuged (Sigma, UK) at 13,000 *× g* for 15 min. The crude DNA in the supernatants was transferred into sterile microcentrifuge tubes and stored at –20 °C until use (total DNA). All primers in this study were synthesized by Sigma-Aldrich Life Science, UK. The primer sequences and expected PCR product sizes are given in Table 4. Unless indicated otherwise, *E. coli* ATCC 25922 was used as a negative control. A 100 bp DNA size marker (NEB, UK) was used in each gel, except where indicated otherwise.

For enteroaggregative (EAggEC) virulence plasmid gene *astA* detection, total DNA (1 μL) was used in a 25 μL reaction mixture that contained 2.5 μL 10 × reaction buffer (ThermoFisher Scientific, UK), 2 μL (2.5 mM) of each of the dNTPs, 1 μL (10 μM) of each primer, 0.125 μL (5 U/μL) Dream*Taq* (ThermoFisher Scientific, UK), and 17.375 μL of molecular biological grade water. *E. coli* H10407 [95] was used as positive control. A programmable C1000TM Thermal cycler (Bio-Rad, UK) was used to carry out the reactions under the following conditions: one cycle of denaturation for 5 min at 94 °C and then 30 cycles comprising 1 min at 94 °C, 1 min at 55 °C, and 1 min at 72 °C, with a final extension of 10 min at 72 °C [37].

For the detection of *β-*lactamase genes, PCR was used to examine for *bla*_SHV_, *bla*_TEM_, *bla*_OXA-1_, and *bla*_CTX-M_ genes [96]. Presence of the *mcr-1* resistance gene was used to determine resistance to colistin sulfate. The reaction mixture was as described above. *E. coli* NCTC 13353 was used as a positive control for CTX, *E. coli* NCTC 13352 as a positive control for TEM, *E. coli* strain A2.5 [38] as a positive control for OXA, and *Klebsiella pneumoniae* NCTC 13368 as a positive control for SHV. *E. coli* NCTC 13846 was used as a positive control for *mcr-1*. Except for *mcr*-1, PCR amplification was one cycle of denaturation for 5 min at 94 °C followed by 30 cycles of 30 s at 94 °C, 30 s at 55 °C, and 1 min at 72 °C, with a final extension of 7 min at 72 °C [38]. The program for *mcr-1* was 10 min at 94 °C followed by 25 cycles of 30 s at 94 °C, 1 min at 58 °C, and 1 min at 72 °C, with a final extension of 10 min at 72 °C. 

For silver resistance, *silA*, *silB*, and *silE* genes were screened [97]. *E. coli* J53 and *E. coli* J53 pMG101 were negative and positive controls, respectively. PCR reactions were carried out under the following conditions: one cycle of denaturation for 3 min at 95 °C followed by 30 cycles of 30 s at 95 °C, 30 s at 54 °C, and 1 min at 72 °C, with a final extension of 5 min at 72 °C. The markers with 100 bp (for *silB* and *silE*) and 1000 bp (*silA*) DNA size were used in each gel (Table 4).

For mercury resistance of *merA* and *merC* genes [99], as well as *intl1* [100] detection, 25 μL reaction mixture contained 2.5 μL 10 × Buffer, 2 μL (2.5 mM) of each of the dNTPs, 0.5 μL (10 μM) of each primer, 0.125 μL (5 U/μL) DreamTaq (ThermoFisher Scientific, UK), 1 μL DNA template, and 18.375 μL of molecular biological grade water. The strains, *E. coli* J53 and *E. coli* J53 pMG101, were used as negative and positive controls, respectively. PCR reactions were carried out under the following conditions: one cycle of denaturation for 5 min at 95 °C followed by 30 cycles of 30 s at 95 °C, 30 s at 67 °C, and 1 min at 72 °C, with a final extension of 5 min at 72 °C.

For copper resistance of *pcoE* gene detection, total DNA (1 μL) was used in a 25 μL reaction mixture that contained 2.5 μL 10 × Buffer, 2 μL (2.5 mM) of each of the dNTPs, 2 μL (10) μM of each primer, 0.125 μL (5 U/μL) DreamTaq (ThermoFisher Scientific, UK), and 15.375 μL of molecular biological grade water. The strains, *E. coli* ED8739 and *E. coli* ED8739 pRJ1004, were as negative and positive controls, respectively. PCR reactions were carried out under the following conditions: one cycle of denaturation for 5 min at 95 °C followed by 30 cycles of 30 s at 95 °C, 30 s at 48 °C, and 1 min at 72 °C, with a final extension of 5 min at 72 °C.

For visualisation of the PCR products, the PCR product (7 μL) with loading buffer (3 μL) was loaded onto a 1.5% w/v agarose gel, containing ethidium bromide (0.4 μg/mL) in 1× TAE running buffer (40 mM Tris-acetate, 1 mM EDTA) and electrophoresed at 100 V for 1 h.

## 5. Conclusions

In this study, we have examined the phenotypic and genotypic resistances to 18 antibiotics and 3 metals (mercury, silver, and copper) of *E. coli* isolated between 2007 and 2010 from pig slaughterhouses in the United Kingdom. The results showed resistances to oxytetracycline, streptomycin, sulphonamide, ampicillin, chloramphenicol, trimethoprim–sulfamethoxazole, ceftiofur, amoxicillin–clavulanic acid, aztreonam, and nitrofurantoin. The top three resistances were oxytetracycline (64%), streptomycin (28%), and sulphonamide (16%). Two strains were resistant to six kinds of antibiotics. Three carried the *bla*_TEM_ gene. Fifteen strains (18.75%) were resistant to 25 µg/mL mercury and five (6.25%) of these to 50 µg/mL; *merA* and *merC* genes were detected in 14 strains. Thirty-five strains (43.75%) showed resistance to silver, with 19 possessing *silA*, *silB*, and *silE* genes. Fifty-five strains (68.75%) were resistant to 8 mM copper or above. Seven contained the *pcoE* gene. Some strains were multi-resistant to antibiotics, silver, and copper. These results indicate the potential for high-risk, multi-drug (metal and antibiotics) resistant strains to enter the food chain and co-selection between antibiotic and metal might exist in *E. coli* isolates, and will aid understanding about the effects of strategies to reduce resistance and mechanisms of antimicrobial resistance (AMR). 

## Figures and Tables

**Table 1:** Antibiotic resistance combinations found in *E.coli* isolates.

Number of Resistance Carried	Total Number of Isolates	Number of Isolates Showing Each Phenotype	Combination of Resistances
6	2	2	AMP *, S10, OT, SXT, S300, C
5	1	1	S10, OT, SXT, S300, C
4	2	1	OT, SXT, S300, C
		1	OT, F, S300, C
3	4	1	AMP, S10, EFT
		1	AMP, S10, OT
		1	S10, OT, S300
		1	AMP, ATM, OT
2	14	9	S10, OT
		4	OT, S300
		1	S10, S300
1	39	30	OT
		7	S10
		2	S300
Intermediate	8	3	AMP
		3	S10
		1	EFT
		1	EFT, S10
0	10	10	Sensitive to all
Total	80	80	

* Antibiotic abbreviations: AMP, Ampicillin; S10, Streptomycin; OT, Oxytetracycline; SXT, Trimethoprim–Sulfamethoxazole; S300, Sulfonamide; C, Chloramphenicol; F, Nitrofurantoin; EFT, Ceftiofur; ATM, Aztreonam.

**Table 2:** Strains with metal resistance genes and/or *β-*lactamase genes and their phenotypic resistances.

Strain		Silver Resistance				Mercury Resistance			Integron	Copper Resistance		Antibiotic Resistance	
		Genotype			Phenotype (400 μM)	Genotype		Phenotype (≥25 μg/mL)		Genotype *pcoE*	Phenotype (≥8 mM)	Phenotype	*β-*Lactamase Genes
ID	Original ID in Reference [37]	*silA*	*silB*	*silE*		*merA*	*merC*		*intl1*				
4	EAggEC7BP4								+ ◊		R∇ (9 mM)	AMP *, S10, OT, SXT, S300, C	*bla*_TEM_
7	EAggEC7BP5								+		R (9 mM)	S10, OT, SXT, S300, C	*bla*_TEM_
9	EAggEC9AP1	+	+	+	R	+	+	R(25 μg/mL)			R (9 mM)	OT	
10	EAggEC8BP5				R	+	+	R(50 μg/mL)	+		R (8 mM)	AMP(I⊗), AMC(I), S10(I), OT, S300, SXT, C	
12	EAggEC10BP1	+	+	+	R						R (8 mM)	S10(I), OT, S300, C, F	
13	EAggEC9BP1	+	+	+	R							AMP, S10, OT	
14	EAggEC5AP4	+	+	+	R	+	+	R(25 μg/mL)	+	+		S10(I), OT, S300, C(I)	
15	EAggEC9AP5	+	+	+	R	+	+	R(25 μg/mL)			R (9 mM)	OT, CIP(I)	
17	EAggEC8AP4	+	+	+	R						R (9 mM)	EFT(I), S10(I)	
21	EAggEC10AP1	+	+	+	R	+	+	R(25 μg/mL)			R (9 mM)	S10(I), OT	
23	EAggEC 11AP3	+	+	+	R	+	+	R(25 μg/mL)			R (9 mM)	S10(I), OT	
24	EAggEC 11AP4	+	+	+	R	+	+	R(25 μg/mL)			R (9 mM)	S10(I), OT	
25	EAggEC9AP2	+	+	+	R	+	+	R(25 μg/mL)			R (9 mM)	S10(I), OT	
27	EAggEC10AP2	+	+	+	R							S10, OT	
38	EAggEC5AO2					+	+	R(50 μg/mL)			R (9 mM)	OT	
41	EAggEC5AO1					+	+	R(50 μg/mL)			R (9 mM)	OT	
48	EAggEC5AO3					+	+	R(50 μg/mL)			R (9 mM)	OT	
50	EAggEC10AO1	+	+	+	R					+	R (8 mM)	S10(I), S300	
57	EAggEC5AS2					+	+	R(50 μg/mL)			R (9 mM)	AMP, ATM, OT	
59	EAggEC5AS1					+	+	R(25 μg/mL)			R (9 mM)	AMP(I), OT	
61	EAggEC10AO3											AMP, AMC, OT, S300, SXT	*bla*_TEM_
62	EAggEC12AO3	+	+	+	R							S10(I), OT	
63	EAggEC12AO4	+	+	+	R					+			
70	EAggEC11AS5	+	+	+	R	+	+	R(25μg/mL)			R (9 mM)	OT	
74	EAggEC14AS2	+	+	+	R					+			
75	EAggEC14AS1	+	+	+	R					+			
77	EAggEC15AS1	+	+	+	R					+	R (9 mM)	S10(I), OT	
78	EAggEC13AO3	+	+	+	R					+	R (10 mM)		

◊: “+” means that gene was detected; ∇: “R” means resistant to the concentration indicated in bracket; * For antibiotic abbreviations, see Table 3. Antibiotic abbreviation means that the strain shows resistance to this antibiotic; ⊗ “I” means the resistance is “intermediate sensitivity” to this antibiotic.

**Table 3:** Antibiotic assay discs, abbreviations, and amount of antibiotic contained in each disc.

Antibiotic Discs	Content	Antimicrobial Group
*β*-Lactams		
Ampicillin (AMP)	10 μg	Penicillins
Amoxicillin–clavulanic acid (AMC)	20/10 μg	Penicillin Combination with Beta-lactamase Inhibitor
Cefotaxime (CTX)	30 μg	Third Generation Cephalosporin
Ceftazidime (CAZ)	30 μg	Third Generation Cephalosporin
Ceftiofur (EFT)	30 μg	Third Generation Cephalosporin
Cefquinome (CFQ)	30 μg	Fourth Generation Cephalosporin
Aztreonam (ATM)	30 μg	Monobactam
Imipenem (IPM)	10 μg	Carbapenem
Aminoglycoside		
Streptomycin (S10)	10 μg	Amino-glycosides
Quinolones		
Ciprofloxacin (CIP)	5 μg	Fluoro-quinolones
Enrofloxacin (ENR)	5 μg	Fluoro-quinolones
Nalidixic acid (NA)	30 μg	Quinolones
Sulphonamide/complex		
Trimethoprim–sulfamethoxazole (SXT)	1.25/23.75 μg	Folate Pathway Inhibitors
Sulfonamide (S300)	300 μg	Folate Pathway Inhibitors
Phenicol		
Chloramphenicol (C)	30 μg	Phenicols
Tetracycline		
Oxytetracycline (OT)	30 μg	Tetracyclines
Nitrofuran derivative		
Nitrofurantoin (F)	300 μg	Nitrofurans

**Table 4:** Polymerase chain reaction (PCR) primers used for detection of *β-*lactamase genes and metal resistance genes.

Oligonucleotide Name	Sequence ^a^	Product Size (Bp)	Reference
*astA*-F	CCATCAACACAGTATATCCGA	111	[98]
*astA*-R	GGTCGCGAGTGACGGCTTTGT		
CTX-M-F	ATGTGCAGYACCAGTAARGTKATGGC	529	[96]
CTX-M-R	TGGGTRAARTARGTSACCAGAAYSAGCGG		
TEM-F	GCGGAACCCCTATTTG	964	
TEM-R	ACCAATGCTTAATCAGTGAG		
SHV-F	TTATCTCCCTGTTAGCCACC	796	
SHV-R	GATTTGCTGATTTCGCTCGG		
OXA-1-F	ATGAAAAACACAATACATATCAACTTCGC	820	
OXA-1-R	GTGTGTTTAGAATGGTGATCGCATT		
*mcr-1*-F	CGGTCAGTCCGTTTGTTC	309	[32]
*mcr-1*-R	CTTGGTCGGTCTGTAGGG		
*merA*-F	ACCATCGGCACCTGCGT	1237	[99]
*merA*-R	ACCATCGTCAGGTAGGGGAACAA		
*merC*-F	CATCGGGCTGGGCTGGGCTTCTTGAG	364	
*merC*-R	CATCGTTCCTTATTCGTGTGG		
*intl1*-F	CCTCCCGCACGATGATC	280	[100]
*intl1*-R	TCCACGCATCGTCAGGC		
*silA*-F	ATGATTGAATGGATTATCCG	3147	[97]
*silA*-R	TTATGACACGCTTTTTTTAT		
*silB*-F	ATGGCTTCTTTAAAGATAAA	1293	
*silB*-R	TCAGTGCCCTGAATGCATAT		
*silE*-F	ATGAAAAATATCGTATTAGC	432	
*silE*-R	TCAGCCTGCACTGAGCATGC		
*pcoE*-F	ATGAATATATTAATCACGAC	450	
*pcoE*-R	TTACCTGGTCGAATACAGCC		

^a^ R is a purine; Y is a pyrimidine; S is G or C.

## Supplementary Materials

The following are available online at https://www.mdpi.com/2079-6382/9/11/746/s1, Table S1: All 81 strains information.
